# A Concise Review on Tissue Engineered Artificial Skin Grafts for Chronic Wound Treatment: Can We Reconstruct Functional Skin Tissue In Vitro?

**DOI:** 10.3390/cells9071622

**Published:** 2020-07-06

**Authors:** Agata Przekora

**Affiliations:** Department of Biochemistry and Biotechnology, Medical University of Lublin, Chodzki 1 Street, 20-093 Lublin, Poland; agata.przekora@umlub.pl; Tel.: +48-81-448-7026

**Keywords:** biomaterials, skin substitutes, nanocomposites, antibacterial skin grafts, epidermal skin grafts, dermal skin grafts, dermo-epidermal skin grafts, mesenchymal stem cells, skin appendages

## Abstract

Chronic wounds occur as a consequence of a prolonged inflammatory phase during the healing process, which precludes skin regeneration. Typical treatment for chronic wounds includes application of autografts, allografts collected from cadaver, and topical delivery of antioxidant, anti-inflammatory, and antibacterial agents. Nevertheless, the mentioned therapies are not sufficient for extensive or deep wounds. Moreover, application of allogeneic skin grafts carries high risk of rejection and treatment failure. Advanced therapies for chronic wounds involve application of bioengineered artificial skin substitutes to overcome graft rejection as well as topical delivery of mesenchymal stem cells to reduce inflammation and accelerate the healing process. This review focuses on the concept of skin tissue engineering, which is a modern approach to chronic wound treatment. The aim of the article is to summarize common therapies for chronic wounds and recent achievements in the development of bioengineered artificial skin constructs, including analysis of biomaterials and cells widely used for skin graft production. This review also presents attempts to reconstruct nerves, pigmentation, and skin appendages (hair follicles, sweat glands) using artificial skin grafts as well as recent trends in the engineering of biomaterials, aiming to produce nanocomposite skin substitutes (nanofilled polymer composites) with controlled antibacterial activity. Finally, the article describes the composition, advantages, and limitations of both newly developed and commercially available bioengineered skin substitutes.

## 1. Introduction to the Problem of Chronic Wounds

**Figure 1:**
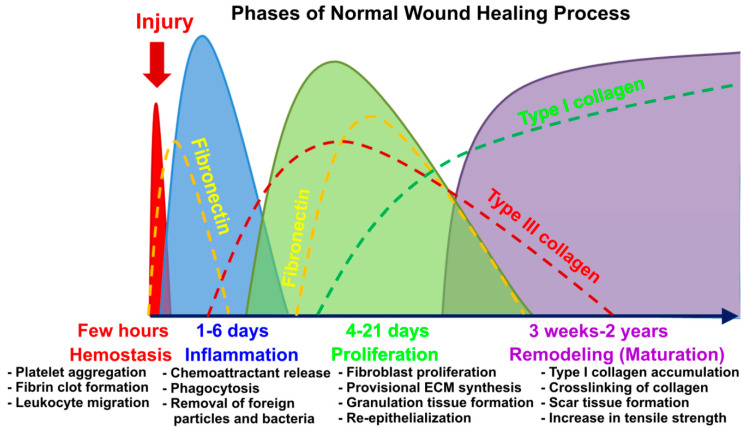
Graphical representation of the four phases of the normal wound healing process.

The skin is the largest organ in the human body, performing a large number of functions crucial for survival. The primary role of the skin is to act as a protective barrier against harmful ultraviolet radiation, chemicals, and pathogenic microorganisms. Other important functions of the skin involve production of vitamin D, regulation of body temperature, and moisture loss control [1,2,3,4]. Wounds occur as a result of thermal or physical injury, causing a break in continuity of the epithelial layer of the skin or mucosa [5]. Wound healing is a complex and multistep process; thus, deep injuries to the skin are a big challenge for doctors. The skin healing process may be divided into four major phases: (1) hemostasis phase (just after injury) aimed at stopping bleeding, (2) the inflammatory phase, (3) the proliferation phase, and (4) the maturation phase (remodeling) (Figure 1) [1,2,5]. After the acute inflammatory phase, which usually occurs during the first four days after injury, inflammatory cells secrete growth factors (GFs), which promote proliferation of vascular endothelial cells and fibroblasts [2]. During the wound healing process, fibrinogen is converted enzymatically by thrombin to fibrin, which has the ability to not only stimulate fibroblast proliferation, but also induce vascularization and tissue repair [6]. Expanded at the wound site, dermal skin fibroblasts produce type III collagen, which gradually replace the fibrin matrix. The next stage of wound healing includes initiation of angiogenesis by endothelial progenitor cells, resulting in the formation of granulation tissue. The late proliferation phase of skin repair is characterized by migration of keratinocytes from the edges of the wound to the granulation tissue, followed by re-epithelialization of the wound. During the maturation phase (skin remodeling), which lasts from a few weeks to even two years, type III collagen is gradually replaced by the stronger type I collagen, which is accumulated and cross-linked to strengthen the wound. Scar tissue formation is also observed [2].

**Figure 2:**
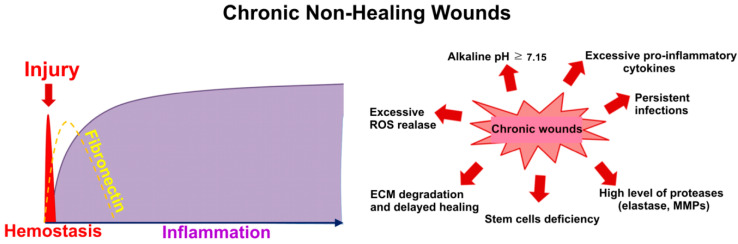
Main features of chronic wounds (ECM: extracellular matrix, MMPs: matrix metalloproteinases, ROS: reactive oxygen species).

Depending on the healing duration, the wound may be classified as acute or chronic. An acute wound usually occurs as a result of sudden skin injury due to an accident or surgical procedure. Importantly, it has the ability to heal at a predictable and relatively short time frame (within 8–12 weeks). Unlike acute wounds, chronic wounds cannot be healed at an expected time frame because they fail to progress beyond the inflammatory phase, precluding proliferation of vascular endothelial cells and fibroblasts as well as collagen matrix deposition. Moreover, it has been demonstrated that chronic wounds are characterized by the imbalance between pro- and anti-inflammatory cytokines and GFs at the wound site, which hinders the wound healing process [7]. All chronic wounds have certain common features: (1) excessive levels of pro-inflammatory cytokines, (2) high level of proteases that cause destruction of extracellular matrix (ECM) and degradation of GFs, (3) excessive release of reactive oxygen species (ROS) by inflammatory cells leading to the oxidative damage of the cells and ECM, (4) deficiency of stem cells hindering skin regeneration, and (5) high risk of persistent infections (Figure 2) [8]. Unhealed chronic wounds may develop after trauma, burns, as a consequence of prolonged pressure on the skin (pressure ulcers), improper function of the venous valves (venous ulcers) or metabolic disorders (diabetic foot ulcers), significantly impacting the comfort of the patients. Therefore, effective treatment of chronic wounds is of high importance.

## 2. Common Therapies for Chronic Wounds

### 2.1. Skin Transplantation

The conventional treatment of chronic wounds includes the use of autografts, allografts (usually taken from cadavers), or xenografts, which are typically harvested from porcine skin [2]. Because of the low risk of immune rejection, autografts are most often used for skin regeneration. Taking into account the anatomical structure of autologous skin grafts, they are classified as epidermal skin grafts (ESGs), split-thickness skin grafts (STSGs), and full-thickness skin grafts (FTSGs) [9,10].

ESGs contain only the epidermal layer of the skin and the graft is harvested under local anesthesia in an outpatient condition. Importantly, the donor site is painless and heals rapidly without scarring. Since ESGs are deprived of dermis, they are recommended for treatment of superficial and small wounds when complete restoration of the skin function is not required [9]. Everts et al. [11] demonstrated that early application of ESGs in combination with other therapies (such as platelet-rich plasma therapy, hyperbaric oxygen treatment, compression therapy) may significantly reduce the surface of a chronic wound, decreasing healing duration.

STSGs contain complete epidermal layer of the skin and part of the dermis. Depending on the thickness of the dermal layer, they are classified as thin, medium, and thick. STSGs are most frequently used in the treatment of large chronic wounds. Although STSG application does not require extraction of full dermis, the procedure of graft collection carries a high risk of complications at the donor site, including scarring, chronic pain, abnormal pigmentation, and infections [9,10]. Nevertheless, Hu et al. [12] demonstrated that a combination of STSG and autologous skin cell suspension (harvested using ReCell^®^ technology) not only improved the healing rate of chronic wounds, but also reduced clinical complications.

FTSGs are made of epidermis and complete dermis, and therefore this type of graft is the only one allowing for full restoration of dermal components [9]. FTSGs require the presence of robust vascular supply at the wound bed, and thus their application is usually limited to smaller wounds. However, Patterson et al. [10] treated extensive skin defect by application of novel commercially available autologous homologous skin construct (AHSC) technology, which allows to use the endogenous cutaneous regenerative potential of the patient to produce full-thickness skin containing all components of the epidermal and dermal layers. Importantly, through this new self-propagating autologous skin graft technology, they achieved successful regeneration of full-thickness autologous hair-bearing skin graft in a critical-sized cutaneous defect, which was created by excision of the scar tissue formed after previous STSG treatment of a burn injury in a 10-year-old boy.

### 2.2. Administration of Anti-Inflammatory and Anti-Oxidant Agents

Apart from the application of skin grafts, the treatment of chronic wounds involves topical application of strong antioxidants or anti-inflammatory agents, which results in the reversal of the chronicity of the wounds [8]. Among various active agents with anti-inflammatory properties, substances occurring in essential oils (EOs) deserve special attention. EOs are widely used in cosmetics, fragrances, and the food industry. Thus, their chemical composition as well as their effects on skin have been well studied for decades. It has been proved that major active substances (e.g., pinene, limonene, thymol, carvacrol) occurring in many EOs may exert strong anti-inflammatory, antioxidant, and antimicrobial effects, which are three crucial issues in chronic wound treatment [13,14,15,16,17]. Da Silva et al. [18] demonstrated that essential oil extracted from Eugenia dysenterica DC leaves had the ability to induce fibroblast migration, possessed anti-inflammatory activity, and promoted angiogenesis in vivo, suggesting that its topical administration may result in faster regeneration of wounds. Mori et al. [19] proved that lavender oil had immunomodulatory activity by upregulation of transforming growth factor-β (TGF-β). As a consequence, topical administration of lavender oil to the wound resulted in the faster development of granulation tissue, enhanced collagen synthesis, and wound contraction. Thymol and carvacrol, which are monoterpenoids abundant in EOs of Thymus vulgaris and Origanum vulgare, are also well known to have a strong immunomodulatory effect (anti-inflammatory and antioxidant) and ability to promote angiogenesis, formation of granulation tissue, and re-epithelialization of the wound [20]. In turn, Saporito et al. [21] developed lipid nanoparticles (NPs) loaded with eucalyptus or rosemary EOs, which significantly enhanced healing of burn wounds in a rat model.

Curcumin is not an EO, but a naturally occurring yellow chemical extracted from the rhizome of Curcuma longa, possessing a strong antioxidant effect and the ability to accelerate chronic wound repair [22,23,24]. Therefore, a large number of curcumin-based topical formulations (including films, emulsions, or hydrogels) for chronic wound treatment have been recently developed [23]. Kant et al. [25] demonstrated that the application of curcumin led to a significantly accelerated wound healing process in diabetic rats by increasing the levels of antioxidant enzymes (superoxide dismutase, catalase, and glutathione peroxidase) and interleukin-10 (IL-10), which is an anti-inflammatory cytokine.

Topical administration of neurotensin (NT), a neuropeptide with immunomodulatory properties, is another strategy for chronic wound treatment. Moura et al. [26] developed collagen dressing loaded with NT, which enhanced skin repair in diabetic wounded mice. They proved that delivery of NT to the wound site significantly decreased the expression of pro-inflammatory cytokines, like tumor necrosis factor-α (TNF-α) and IL-1β, as well as increased fibroblast migration and collagen deposition. However, in contrast to the NT-loaded collagen dressing, the application of NT alone reduced scar formation.

The reversal of wound chronicity and the subsequent accelerated healing process may also be achieved by topical application of therapeutic anti-TNF-α antibodies. Sandhu et al. [27] used Adalimumab (anti-TNF monoclonal antibody) for the treatment of refractory ulcerated necrobiosis lipoidica and showed complete re-epithelialization of the ulcers within 28 weeks.

### 2.3. Administration of Growth Factors and Protease Inhibitors

It has been demonstrated that chronic wound fluid contains significantly increased levels of elastase and matrix metalloproteinases (MMPs), including MMP-1, MMP-2, MMP-8, MMP-9, and MMP-13 [28,29]. Chronic wounds are also characterized by reduced levels of various GFs important for wound healing, such as transforming growth factor-β (TGF-β), epidermal growth factor (EGF), fibroblast growth factor (FGF), vascular endothelial growth factor (VEGF), and platelet-derived growth factor (PDGF) [30]. Thus, topical administration of the mentioned GFs on the wound area appears to be a rational strategy for chronic wound treatment. Nevertheless, high levels of active proteases at the wound site cause degradation of GFs; thus, most therapies involving administration of GFs alone have failed. Therefore, Stacey [28] recently developed a protocol for topical application of GFs in combination with protease inhibitors to overcome the problem of GF degradation and to increase the effectiveness of chronic wound therapy. A great variety of potential protease inhibitors were identified and demonstrated to be effective in chronic wound treatment. Doxycycline is an antibiotic revealing an anti-inflammatory effect through the inhibition of MMPs at the wound site [24]. Promogran™ is a porous and highly absorbent wound dressing made of collagen and oxidized regenerated cellulose, which, on contact with the wound exudate, becomes a soft gel. In turn, the formed gel has the ability to make inactive elastase and MMPs as well as to bind to GFs, preventing them from degradation [31]. Other protease inhibitors used for chronic wound treatment involve SB-3CT that inhibits MMP-2 and MMP-9 [32] or (R,S)-ND-322 [33] and (R,S)-ND-336 [34], which are water-soluble selective MMP-9 and MMP-8 inhibitors.

## 3. Modern Approaches to Chronic Wound Treatment

### 3.1. Topical Delivery of Adult Stem Cells’ Suspension

The endogenous population of adult stem cells play a pivotal role in cutaneous wound healing. It has been demonstrated that bone marrow-derived stem cells (BMDSCs) have the ability to migrate to the injured tissues, where they proliferate and differentiate into the required cell type, including fibroblasts [35]. It was estimated that up to 20% of fibroblasts at the wound site may originally derive from BMDSCs [36]. Similarly, adipose tissue-derived stem cells (ADSCs) can differentiate and regenerate damaged skin tissue, whereas re-epithelialization of the wound occurs as a consequence of differentiation of interfollicular and hair follicle bulge epithelial stem cells toward keratinocytes [35]. Revascularization of the injured skin is the effect of either proliferation of endothelial cells in existing blood vessels (angiogenesis) or differentiation of endothelial progenitor cells (vasculogenesis). Nevertheless, new blood vessels are formed at the wound site, mainly through the angiogenesis mechanism [37], whereas the major role of endothelial progenitor cells is to secrete pro-healing GFs [35,38].

Taking into account the crucial role of adult stem cells in the wound healing process, another therapy for chronic wounds includes direct delivery of healthy functional mesenchymal stem cells (MSCs) to overcome their deficiency at the wound site and to achieve better healing rate. Although MSCs are multipotent progenitor cells known to have the ability to differentiate into mesenchymal tissues (such as bone, tendon, and cartilage), their role in the skin healing process is related to paracrine function and synthesis of various GFs and cytokines that are essential for cell migration, proliferation, as well as controlling the inflammatory phase during skin regeneration. Importantly, MSCs not only respond to inflammation by upregulation of synthesis of anti-inflammatory cytokines, but also promote angiogenesis by VEGF release [8]. Moreover, it was demonstrated that delivery of BMDSCs or ADSCs to the wound area significantly reduces scar formation [7]. Furthermore, MSCs do not have surface antigens that would typically engender a foreign body reaction, thus allogenic MSCs may be safely used in clinical applications [8].

ADSCs are isolated from stromal vascular fraction (SVF), which is typically obtained by digestion of adipose tissue with collagenase, followed by few cycles of centrifugation [39,40]. The SVF is a mixture of stem cells (10–30% of ADSCs, pericytes, hematopoietic stem cells), mature cells (fibroblasts, muscle cells, endothelial cells, and blood cells), as well as various cytokines and GFs, and thus it reveals strong immunomodulatory properties and the ability to promote skin regeneration, which is comparable or even better than activity recorded for ADSCs [41,42,43]. Atalay et al. [40] applied SVF for the treatment of burn wounds in a rat model. They observed that intradermal administration of SVF into the deep partial-thickness burns accelerated wound healing by increasing fibroblast proliferation, vascularization, and reducing inflammation. Sun et al. [44] developed injectable ECM/SVF-gel for stem cell therapy of the wounds. They demonstrated, using a nude mouse excisional wound healing model, that ECM/SVF-gel had the ability to promote vascularization by secretion of angiogenic factors. Similarly Deng et al. [45] applied an autologous ECM/SVF gel to treat chronic wounds of patients in clinics and showed an accelerated healing process, which resulted from the immunomodulatory effect of SVF, increased collagen deposition, and improved vascularization.

### 3.2. Skin Tissue Engineering

**Figure 3:**
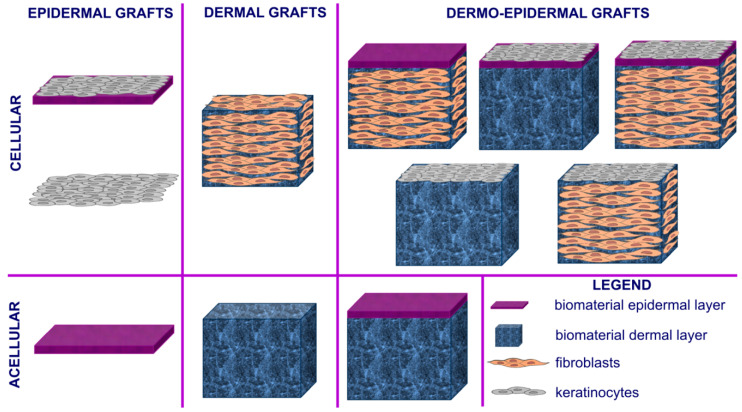
Different variants of tissue-engineered artificial skin grafts.

The use of autologous skin grafts is the most common approach in the treatment of chronic wounds. However, in the case of deep and/or large wounds or with extensive severe burns, the use of autografts is limited, and either allogeneic (from cadaver) or xenogeneic skin grafts are used for transplantation. Nevertheless, the use of allogeneic/xenogeneic tissue carries a high risk of graft rejection, limiting their clinical applications [46]. To overcome deficiency of donor skin graft supplies as well as skin allo/xenograft rejections, modern treatment includes skin tissue engineering aiming to produce bioengineered biomaterial-based artificial skin grafts [2,46]. This advanced strategy for wound regeneration aims to fabricate skin substitutes acting as bioactive wound dressings, facilitating the function of the wound (not just covering it). Therefore, the main roles of bioengineered skin grafts is to supply oxygen (by being oxygen permeable), keep the wound from dehydration, promote healing, and prevent infections [2,46,47,48]. Depending on the type of biomaterial used for the production of artificial skin grafts, they may function as skin equivalents providing temporary wound covers or permanent skin substitutes. When the biomaterials are pre-seeded or have cells incorporated within their matrix, they are classified as cellular artificial skin grafts, whereas biomaterials without or deprived of cells are defined as acellular artificial skin grafts [2,46]. Depending on their anatomical structure, similarly to autologous skin transplants, artificial skin substitutes may be categorized as epidermal, dermal, or dermo-epidermal (Figure 3) [2,49,50].

**Figure 4:**
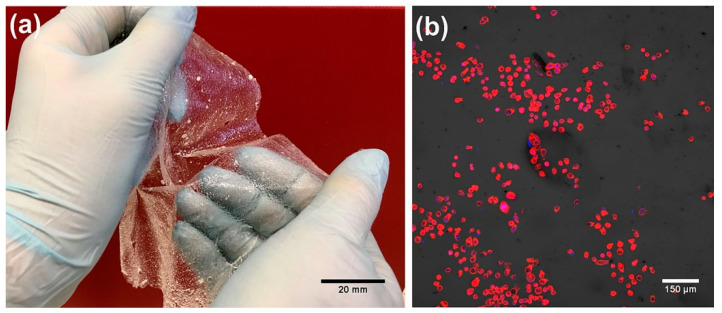
Epidermal skin graft: (**a**) image presenting a thin chitosan/agarose membrane (produced according to Polish patent application no. P.430458 [52,53] for regenerative medicine application as epidermal skin substitute; (**b**) confocal laser scanning microscope (CLSM) image presenting human epidermal keratinocytes stained with AlexaFluor635-Phalloidin (red fluorescence of cytoskeleton) grown on the surface of the chitosan/agarose membrane that was visualized by application of Nomarski contrast.

Epidermis is non-vascular tissue; thus, the primary role of epidermal skin constructs is to promote re-epithelialization and cover the wound bed, protecting the wound against dehydration and infections. Modern epidermal skin grafts usually have a form of thin membrane, which supports the adhesion and proliferation of keratinocytes (Figure 4). Autologous keratinocytes are isolated from patient tissue biopsy, seeded onto the surface of the biomaterial-based membrane, and expanded under in vitro conditions to form a thin sheet of cellular epidermal layer of the skin. For instance, Paggiaro et al. [51] used glycerolated human amniotic membrane for the fabrication of cellular epidermal graft seeded with keratinocytes. Nevertheless, it usually takes as long as 2–3 weeks to generate autologous epidermal graft, which is the main disadvantage of cellular epidermal substitutes [49,50]. However, it has been shown that the application of natural biopolymers as membranes for cell cultivation, like fibrin, chitosan or hyaluronic acid (HA), significantly reduces the time needed for the production of cellular epidermal skin grafts, since these biomaterials are known to promote the migration and proliferation of cultured keratinocyes [49].

**Figure 5:**
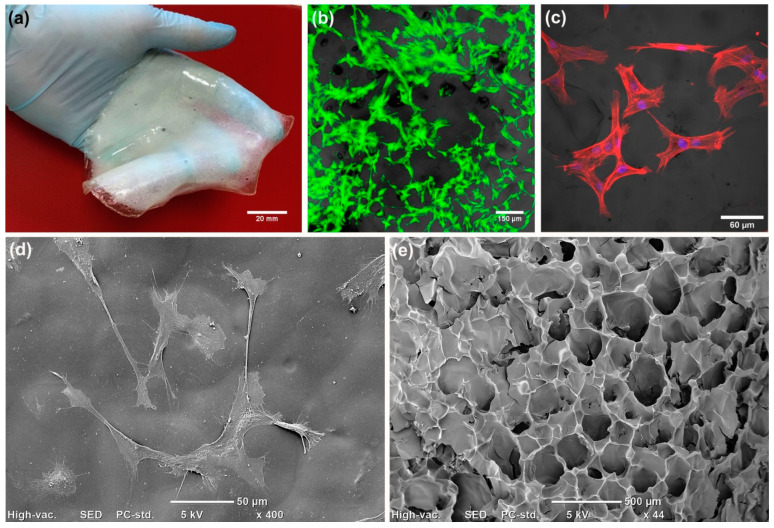
Dermal skin graft: (**a**) image presenting 2-mm thick chitosan/curdlan film (Polish patent application no. P.430456 [59]) for regenerative medicine application as dermal skin substitute; (**b**) CLSM image presenting human skin fibroblasts stained with calcein-AM (green fluorescence of viable cells) and propidium iodide (red fluorescence of dead cells) grown on the surface of the chitosan/curdlan film that was visualized by application of Nomarski contrast; (**c**) CLSM image presenting human skin fibroblasts stained with AlexaFluor635-Phalloidin (red fluorescence of cytoskeleton) and DAPI (blue fluorescence of nuclei) grown on the surface of the chitosan-based thin membrane that was visualized by application of Nomarski contrast; (**d**) scanning electron microscope (SEM) image presenting well-attached human skin fibroblasts on the surface of the chitosan hydrogel; (**e**) SEM micrograph presenting porous foam-like chitosan-based wound dressing (Polish patent application no. P.430455 [60]) for regenerative medicine application.

The dermis layer of the skin is made of fibroblasts and ECM composed of type III collagen (upper papillary region) and type I collagen (lower reticular region) [49]. To reduce fabrication costs, dermal skin substitutes are often made of biomaterial-based matrix without any cells incorporated (acellular grafts). Thus, the primary role of acellular dermal constructs is to act as a framework for fibroblasts and endothelial cells’ migration and infiltration upon graft transplantation into the living organism [49,50]. Nevertheless, there also are numerous cellular dermal grafts that may be pre-seeded with skin fibroblasts in vitro before transplantation to accelerate healing (Figure 5a–d)). Since dermis is a highly vascularized tissue, dermal skin grafts for chronic wound treatment should have high macroporosity (diameter of pores >100 µm) with a high share of open and interconnected pores, which are essential for good cell migration and new blood vessels formation (Figure 5e). Moreover, the porous structure of the dermal grafts allows for transport of nutrients and growth factors, which are crucial for cell proliferation and survival [46].

Dermo-epidermal skin substitutes are the most advanced among all types of artificial grafts. They are made of both layers of the skin (dermal and epidermal), ideally mimicking natural tissue. Artificial dermo-epidermal grafts are often prepared by culture of keratinocytes on the surface of the dermal layer with or without fibroblasts incorporated. Another type of dermo-epidermal grafts involves bilayered biomaterials composed of acellular or cellular dermal layer covered by thin polymer-based membrane acting as the acellular epidermal layer [54,55]. Nevertheless, constructs containing both types of the cells allow keratinocyte-fibroblast crosstalk through the release of GFs and cytokines, which is very important for a fast healing process and promotion of re-epithelialization [49,50]. To produce functional dermo-epidermal constructs, researchers frequently use various advanced techniques like 3D bioprinting [56,57] or electrospinning [58].

#### 3.2.1. Cells Used for Cellular Skin Graft Production

**Figure 6:**
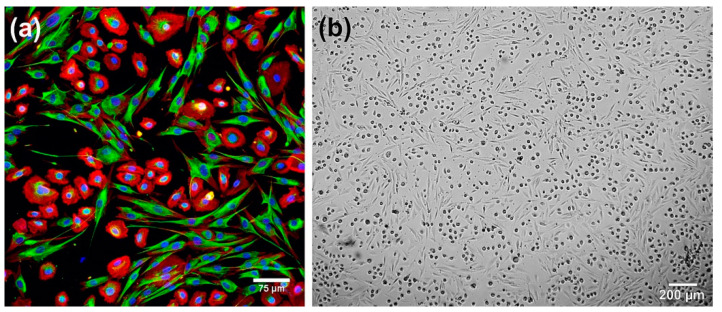
Co-culture of human skin fibroblasts and keratinocytes: (**a**) CLSM image (fibroblasts show blue fluorescence of nuclei, red fluorescence of actin filaments, and green fluorescence of vimentin filaments, whereas keratinocytes show blue fluorescence of nuclei and red fluorescence of actin filaments); (**b**) phase-contrast image showing co-culture of skin cells (fibroblasts reveal spindle-shaped morphology, whereas keratinocytes are visible as round cells).

Cellular skin substitutes are produced using fibroblasts (dermal grafts), keratinocytes (epidermal grafts) or both fibroblasts and keratinocytes (dermo-epidermal grafts). Importantly, it was observed that co-culture systems have advantages over monocultures since fibroblasts have the ability to promote keratinocyte growth and differentiation by either GF release or direct cell-cell contact, whereas GFs secreted by keratinocytes stimulate fibroblast proliferation (Figure 6) [2,49,50].

Fibroblasts and keratinocytes may be obtained by either direct isolation from a skin biopsy or differentiation of stem cells toward mature skin cells. Many research articles present fabrication of tissue-engineered skin grafts using fibroblast or keratinocyte cell lines, e.g., BJ-5ta cells, which are human telomerase reverse transcriptase (hTERT)-immortalized human newborn foreskin fibroblasts [61]; HFF-1 cells, which are normal human newborn foreskin fibroblasts [62]; N/TERT-1 cells, which are hTERT-immortalized human newborn foreskin keratinocytes with spontaneous loss of p16 expression [61,63]; HaCaT cells, which are spontaneously transformed aneuploid immortal keratinocytes from adult human skin [62]; or HEK001 cells, which are human adult epidermal keratinocytes transformed with human papillomavirus 16 [64]. Nevertheless, it is known that cell lines may reveal a completely different gene expression compared to primary cells [65,66], and thus primary cultures should be preferentially used for both skin graft production and biocompatibility testing of new biomaterial-based skin substitutes. Indeed, most cellular artificial skins were developed using either isolated primary fibroblasts and/or keratinocytes [67,68,69,70] or primary culture of skin cells purchased from the cell culture bank [71,72]. The main disadvantage of the use of isolated primary cells is their low proliferation potential, making it difficult to obtain a sufficient number of cells for cellular skin graft production [49].

Rapidly proliferating embryonic stem cells (ESCs), which may be differentiated toward fibroblasts [73] and keratinocytes [74], appear to be good source of skin cells for bioengineered graft production. Nevertheless, the use of embryonic cells is limited due to serious ethical concerns and high risk of teratoma development. Thus, adult MSCs (including BMDSCs and ADSCs) are preferentially used as optimal source of skin cells for living skin construct generation [49,75]. However, ADSCs are most often used in tissue engineering applications due to high availability and easy access to adipose tissue as well as the less-invasive procedure of tissue harvesting compared to BMDSCs [66]. Good alternative to MSCs is application of induced pluripotent stem cells (iPSCs), which are characterized by similar proliferation capacity to ESCs, but their use is ethically approved as iPSCs are obtained from somatic cells by introducing appropriate transcription factors [76,77]. Nevertheless, the development of iPSCs is still a new technology, which carries high risk of carcinogenesis, and thus their clinical use is currently not allowed [49].

#### 3.2.2. Biomaterials Used for Skin Graft Production

Artificial skin grafts are usually fabricated using either natural polymers, like collagen, gelatin, chitosan, fibrin, and HA or synthetic polymers, e.g., polyethyleneglycol (PEG) or polylactic-co-glycolic acid (PLGA) [78,79,80,81,82]. Naturally occurring materials (e.g., fibrin, fibronectin, collagen, HA, chitosan, and glycosaminoglycans–GAGs), which have the ability to restore the physiological functionality of the ECM, are preferentially used for the development of artificial skin substitutes because they have been reported to provide the best healing process for chronic wounds [5,83,84,85]. Mentioned biomaterials possess some important features like low antigenicity, good biodegradability, low toxicity, as well as low risk of chronic inflammatory responses and rejection [3,46]. Skin substitutes are also often produced in combination with growth factors and antimicrobial agents to enhance the wound healing process and protect against infections [86,87].

Among all natural polymers, collagen, chitosan, and HA are most often used in skin tissue engineering. Especially type I collagen, which is abundant protein in the dermis layer of the skin, is a common component of bioengineered artificial grafts [49]. HA, which is a linear polysaccharide made of β-1,4-linked d-glucuronic acid and β-1,3-linked *N*-acetyl-d-glucosamine disaccharide units, also occurs in the ECM and thus is used for the production of artificial skin grafts [46]. In turn, chitosan (polysaccharide generally obtained by deacetylation of chitin occurring in the exoskeleton of crustaceans) is widely selected for the production of skin grafts due to its structural similarity to GAGs of ECM, non-toxicity, biodegradability, inherit antibacterial properties, as well as ability to support fibroblast and keratinocyte adhesion and growth [88,89,90].

Yu et al. [86] produced by freeze-drying method a sponge-like wound dressing made of HA combined with collagen, which was enriched with EGF or FGF. They demonstrated using in vitro cellular models that FGF-enriched wound dressing induced increased VEGF and hepatocyte growth factor (HGF) production by fibroblasts, whereas an EGF-enriched wound dressing had the ability to promote the proliferation of keratinocytes. Maarof et al. [68] developed an acellular dermal collagen skin graft enriched with conditioned medium harvested from human dermal fibroblast culture, which had the ability to accelerate the healing rate and induce complete re-epithelialization of full-thickness wounds in a mouse model without any signs of rejection. Matsumine et al. [91] applied the FGF-impregnated collagen-gelatin sponge for the treatment of patients with acute full-thickness skin defects, achieving wound closure in a short period of time without any complications. Shimizu et al. [92] developed a wound dressing made of HA spongy sheet containing arginine, vitamin C derivate, and EGF, which promoted granulation tissue formation associated with angiogenesis in full-thickness skin defect animal models (rat and diabetic mouse). Lu et al. [84] prepared a chitosan/gelatin sponge enriched with vitamin C and cross-linked with tannin acid, which accelerated healing of full-thickness skin wounds in rabbits and revealed antibacterial properties against *Escherichia coli* and *Staphylococcus aureus* with low toxicity. Mahmoud et al. [87] fabricated norfloxacin-loaded collagen/chitosan sponges, which enhanced the regeneration of full-thickness skin wounds in a rat model without any side-effects. Won et al. [72] developed an innovative cellular dermal skin graft that was prepared using freeze-dried and powdered skin decellularized extracellular matrix (dECM), which had all functional proteins of the ECM preserved, including collagen, GAGs, and GFs. Powdered skin dECM was used for the preparation of bionk that was subsequently 3D bioprinted with human dermal fibroblasts to obtain functional cellular dermal construct. Arasteh et al. [93] produced a bilayered skin substitute by electrospinning of silk fibroin on the human amniotic membrane, which had the ability to accelerate skin regeneration of full-thickness skin wounds in mice by reduction of inflammation, improvement of neovascularization, and limitation of scarring.

Due to the low mechanical stability of biological polymers, synthetic materials have been recently more often used for skin graft production. However, synthetic polymers are deprived of natural biological epitopes that interact with cell receptors or adhesion proteins, supporting cell attachment. Therefore, synthetic hydrogels are usually combined with natural polymers to produce bio-functional composite material with good mechanical properties and high biocompatibility [46,81]. Chandrasekaran et al. [94] fabricated, by electrospinning technique, a nanofibrous scaffold made of poly(l-lactic acid)-co-poly(ε-caprolactone) (PLACL) and gelatin, which supported fibroblast proliferation and deposition of collagen under in vitro conditions. Similarly, Sridhar et al. [95] developed electrospun PLCAL/silk fibroin dermal substitute enriched with vitamin C and tetracycline hydrochloride, which supported fibroblast proliferation and promoted increased collagen deposition in vitro. Sobhanian et al. [96] applied electrospinning for the production of collagen-grafted poly(vinyl alcohol)/gelatin/alginate skin substitute with improved biocompatibility and wound healing properties. Miguel et al. [97] developed acellular dermo-epidermal skin construct by electrospinning of the epidermal layer made of poly(ε-caprolactone)(PCL) and silk sericin on the top of a 3D printed chitosan/sodium alginate hydrogel that served as a dermal layer. Haldar et al. [98] produced a highly biocompatible trilayered acellular dermo-epidermal PCL/gelatin scaffold exhibiting similar architecture and mechanical properties as the actual epidermis, dermis, and hypodermis layers of the skin. Other examples of different types of skin grafts made of natural polymers and their composites with synthetic materials are presented in Table 1.

#### 3.2.3. Nanocomposite Artificial Skin Grafts with Antibacterial Properties

Chronic wounds are characterized by the presence of persistent infections. Most chronic wound pathogens, such as *S. aureus* and *Pseudomonas aeruginosa*, have the ability to form bacterial biofilms. Importantly, bacteria occurring within the mature biofilm are characterized by up to 500 times higher resistance to antibiotics than planktonic cells (freely living). Nevertheless, topical delivery of antibiotics (due to high risk of antibiotic resistance, superinfection, impaired healing, and delayed hypersensitivity reaction) is not recommended unless critical bacteria colonization is recognized. Furthermore, systemic antibiotics should be used only in the case of sepsis, abscess formation, cellulitis, osteomyelitis, or lymphangitis [108]. Therefore, there is a huge need to develop antibiotic-free biomaterial-based artificial skin grafts, revealing resistance to bacterial colonization and antibacterial activity by controlled and sustained release of antibacterial agents (but not antibiotics), and thereby protecting against invasive infections in the chronic wound microenvironment. As incorporation of antibiotics into biomaterial structure is not recommended, there is a growing trend in the engineering of biomaterials to fabricate nanocomposite artificial skin grafts and wound dressings made of polymer matrix reinforced with antimicrobial nanoparticles (nanofilled polymer composites).

Since silver (Ag^+^) metal ions are known from their broad antimicrobial spectrum against various bacteria and fungi, silver nanoparticles (nAg) have been the most extensively used for reinforcement of polymer matrix for chronic wound healing applications [85,109]. Pérez-Díaz et al. [110] developed chitosan gel reinforced with nAg that had the ability to prevent bacterial biofilm formation and kill *S. aureus* and *P. aeruginosa* bacteria in established biofilm. Moreover, developed chitosan/nAg formulation revealed reduced cytotoxicity compared to the silver sulfadiazine that was used in the experiments as a reference treatment. Anisha et al. [111] produced an antibacterial sponge-like wound dressing composed of chitosan, HA, and nAg for diabetic foot ulcers infected with drug-resistant bacteria. They demonstrated high antibacterial efficiency (with low cytotoxicity at lower concentrations of nAg) of the wound dressing against *E. coli*, *S. aureus*, methicillin-resistant *S. aureus* (MRSA), *P. aeruginosa*, and *Klebsiella pneumonia*. Nevertheless, chemical synthesis of nAg is limited by high costs, energy consumption, and environmental toxicity. Thus, non-polluting green synthesis of nAg (based on plant extracts) has gained popularity due to obvious environmental reasons. Plant extracts, which are rich in many alkaloids, phenols, amino acids, carbohydrates, and proteins, may be effectively involved in the reduction and stabilization of Ag ions in nAg [112]. Raghavendra et al. [113] used natural carbohydrates (gum acacia and gaur gum) as effective reductants during green synthesis of nAg from AgNO_3_. The produced nAg were subsequently incorporated within cellulose fibers to form biomaterials with antibacterial activity against *E. coli*. In another study, Arya et al. [114] synthesized nAg using phenolic compounds from *Prosopis juliflora* leaf extract for the reduction of Ag^+^ to Ag^0^. Produced by green synthesis, nAg showed antibiofilm activity against *Bacillus subtilis* and *P. aeruginosa* as well as the ability to promote wound healing in a mouse model, whereas Sood et al. [115] employed *Ocimum sanctum* for the green synthesis of nAg that were incorporated into Carbopol^®^ 974P NF gel to form a nanocomposite with antibacterial activity against *S. aureus*, *E. Coli*, and *P. aeruginosa*. Moreover, the developed nanocomposite gel promoted faster wound healing in partial thickness burns in a rat model.

Similarly to Ag^+^ ions, zinc (Zn^2+^) metal ions also possess a wide spectrum of antimicrobial activity. Therefore, many antibacterial nanocomposites for chronic wound treatment have been developed using zinc oxide nanoparticles (nZnO). Nevertheless, nZnO are known to induce a time- and concentration-dependent cytotoxicity against eukaryotic cells due to the rapid dissolution of nZnO in the aqueous environment. Importantly, it was noticed that particle size of nZnO has a great impact on its cytotoxicity and antibacterial activity. According to the available literature, it was suggested that nZnO with particle size of < 100 nm exhibited efficient antibacterial activity with reduced toxicity against eukaryotic cells. Importantly, it was also observed that the released Zn^2+^ ions from nZnO may positively affect keratinocyte migration, promoting re-epithelialization [109]. Kumar et al. [116] developed a chitosan/nZnO nanocomposite with antibacterial activity against *P. aeruginosa, Staphylococcus intermedius*, and *Staphylococcus hyicus*. The produced nZnO-based nanocomposite was non-toxic against human dermal fibroblasts and accelerated wound healing and re-epithelialization in a rat model, whereas Shao et al. [117] used *Barleria gibsoni* aqueous leaf extract for green synthesis of nZnO that were embedded into Carbopol^®^ 974P NF gel. The resultant nanocomposite formulation showed efficient antibacterial activity against *S. aureus*, *E. Coli*, and *Proteus vulgaris*, non-toxicity against mouse fibroblasts, and the ability to promote wound healing in a burn rat model.

Nanosized titanium dioxide particles (nTiO_2_) are another type of nanofiller that has been used for the development of antibacterial skin grafts and wound dressings. It has been demonstrated that addition of nTiO_2_ into the polymer matrix not only improves its mechanical properties, but also provides antibacterial activity against various Gram-positive and Gram-negative bacteria. However, nTiO_2_ particles were also shown to induce inflammatory responses, cytotoxicity, and ROS generation in a variety of cell types in vitro. The cytotoxicity effect of nTiO_2_ may be overcome by controlled and slow release of the titanium ions from the composite biomaterial, providing antibacterial protection and accelerated wound healing [109,118]. For instance, Archana et al. [118] developed chitosan/pectin/nTiO_2_ nanocomposite revealing antibacterial activity (tests on *E. coli, S. aureus, P. aeruginosa, B. subtilis)*, non-toxicity against mouse fibroblasts, and accelerated wound healing in a rat model. Similarly, Peng et al. [119] produced antibacterial chitosan/nTiO_2_ nanocomposite that provided accelerated wound healing and steady immunological response in a rat model. Whereas, Woo et al. [120] prepared bilayered nanocomposite for full-thickness wound healing that was composed of chitosan/nTiO_2_ upper layer and a sub-layer made of ECM sheet derived from human adipose tissue. The top layer of the nanocomposite exhibited antibacterial activity against *E. coli* and *S. aureus*, whereas the bottom layer promoted wound healing, re-epithelialization, and angiogenesis in a rat model. More examples of antibacterial nanocomposites for potential use as artificial skin grafts and wound dressings are presented in Table 2.

#### 3.2.4. Reconstruction of Skin Appendages, Pigmentation, and Nerves Using Artificial Skin Grafts

The main limitation of artificial skin grafts is their ability to generate only the epidermal, dermal, and hypodermal layers of the skin. Dermis and epidermis are usually reconstructed using biomaterials seeded with fibroblasts and keratinocytes [67,71], whereas the hypodermis is formed by incorporation of ADSCs within the biomaterial structure [75]. Most of the developed artificial skin substitutes fail to reconstruct nerves and skin appendages, like hair follicles, sweat glands, and sebaceous glands. Therefore, application of artificial grafts often results in the lack of hairs and sweat glands as well as loss of skin pigmentation and sensation, seriously affecting the quality of life of the patients. Since skin appendages and nerves play a pivotal role in the regulation of chemical, physical, and biological functions of the skin, complete cutaneous regeneration after artificial skin graft transplantation should be considered when the recovery of skin sensory and thermoregulatory functions is observed. To achieve this goal, biomaterial-based artificial skins may be combined with stem cells and bioactive molecules to induce formation of appendages and nerves in order to restore temperature, pain, and touch perceptions. Reconstruction of sweat glands, pigmentation, hairs, and nerves to improve patients’ quality of life is currently the greatest challenge in the field of skin tissue engineering and regenerative medicine. Therefore, many studies have been performed to reconstruct skin appendages and nerves with the use of various types of stem cells and differentiation inducers.

Importantly, it has been demonstrated that the hair follicle bulge region may be a good source of stem cells capable of differentiation toward epidermis and different skin appendages, including hair follicles, sebaceous glands, and sweat glands [138]. However, other types of stem cells may be also used for the generation of skin appendages. Kataoka et al. [139] transplanted BMDSCs harvested from adult green fluorescent protein (GFP)-transgenic mice into skin defects in a nude mouse model and detected (within three weeks) the presence of GFP-positive cells in the epidermis, hair follicles, sebaceous glands, and dermis. Similarly, Fang et al. [140] transplanted porcine BMDSCs labeled with BrdU into a porcine skin and observed their transdifferentiation into BrdU-positive sebaceous duct cells. In another study, Xu et al. demonstrated that keratinocyte growth factor (KGF) induced differentiation of human umbilical cord-derived mesenchymal stem cells (hUC-MSCs) toward sweat gland-like cells (SGCs). Furthermore, they proved that treatment of burned paws of mice with SGCs reconstructed sweat glands already after 21 days [141].

Despite many successful reconstructions of skin appendages achieved by transplantation of stem cells are reported in the literature, several attempts have been undertaken to develop artificial skin grafts capable of restoration of hair and sweat glands. Table 3 summarizes attempts to generate artificial skin grafts capable of reconstruction of skin appendages, pigmentation, and nerves. Mohd Hilmi et al. [107] incorporated human fibroblasts and hair follicle stem cells into the chitosan skin substitute that was subsequently transplanted into a full-thickness wound in an irradiated rat model with impaired healing and hair loss. They observed that the applied cellular skin graft accelerated skin regeneration and was able to supply viable follicle stem cells into the irradiated wound. Sriwiriyanont et al. [142] reconstructed hair follicles in an athymic nude mouse model using collagen/glycosaminoglycan scaffolds seeded with murine dermal papilla cells, human fibroblasts, and human keratinocytes, proving that dermal papilla cells play a crucial role in the induction of hair morphogenesis. Meanwhile, Abaci et al. [143] demonstrated that the induction of hair follicle differentiation in artificial skin graft may be achieved by co-culturing human keratinocytes, fibroblasts, and dermal papilla cells and by mimicking the physiological 3D organization of cells in the hair follicle microenvironment.

Patients with extensive burns frequently suffer from loss of hair and sweat glands that are responsible for thermoregulation. While the lack of hair is mainly aesthetic issue, the loss of sweat glands may significantly reduce the quality of life. Thus, reconstruction of sweat glands is of high importance. Shu et al. [144] seeded human eccrine sweat gland cells (SGCs) onto Matrigel^TM^™ matrix (composed of ECM proteins including laminin, type IV collagen, heparan sulfate proteoglycans, entactin/nidogen) and observed the formation of tubular-like structures (made of 1-2 layers of epithelial cells) in vitro, resembling the secretion part and the duct part of the eccrine sweat gland occurring in vivo. Similarly, Li et al. [145] used Matrigel™ matrix with embedded human SGCs to reconstruct sweat gland in an athymic nude mouse model. They proved that restored 3D structures showed expression of proteins related to sweat secretion and absorption as well as markers typical of sweat glands. Whereas Huang et al. [146] used EGF-loaded gelatin microspheres for delivery (by injection) of SGCs into the superficial layer of the bioengineered skin construct, which was generated under in vitro conditions by seeding human keratinocytes on top of the fibroblasts-containing collagen-Matrigel™ matrix. They demonstrated that SGCs delivered into the bioengineered skin graft had the ability to differentiate toward a sweat gland-like structure in vitro.

Transplantation of artificial skin substitutes often causes abnormal pigmentation at the grafting site, which is a huge aesthetic issue, especially in the case of patients with dark complexion. The only possibility to restore pigmentation is transplantation of melanocytes. Ng et al. [147] developed 3D biomimetic dermal skin construct that was prepared by culturing keratinocytes and melanocytes on top surface of the cellular dermal layer made of collagen with embedded fibroblasts. After long-term culture in vitro (39 days), the 3D bioprinted graft showed uniform skin pigmentation and exhibited similarities with native skin tissue in term of the presence of stratified epidermal layers and the continuous layer of basement membrane. Interestingly, spontaneous repigmentation may be achieved unintentionally by transplantation of “passenger” melanocytes along with cultured keratinocytes. Harriger et al. [148] generated artificial skin grafts by culturing autologous keratinocytes and fibroblasts on the collagen/glycosaminoglycan growth substrate. The prepared skin constructs were transplanted into excised, full-thickness burns of the patients. Apart from improved healing, spontaneous repigmentation of healing skin construct due to the presence of “passenger” melanocytes was observed 2 months after grafting.

To restore sensory functions of the skin, it is necessary to induce neuroregeneration process at the site of grafting. It is known that repair of the skin nerves may occur by either stem cell differentiation or extension of healthy axons [138,149]. Therefore, application of artificial skin grafts pre-seeded with stem cells may be a rational strategy for promotion of neuroregeneration. Importantly, it was demonstrated that skin-derived precursor stem cells, which may be easily obtained by skin tissue biopsy, have great potential to induce regeneration of skin sensory nerves due to their ability to differentiate toward nerve and glial cells in vitro [149]. Nevertheless, neuroregeneration of the skin may be affected by several factors. It was observed that in the case of severe burns, fibroblasts show enhanced proliferation and tendency to accumulate around the nerve endings, hindering skin neuroregeneration [150]. Furthermore, it was demonstrated that after artificial skin transplantation, nerve regeneration highly depends on the newly formed blood vessels that supply nutrition and neurotrophic factors (NTFs) for skin nerve cells as well as inhibit elevated fibroblast proliferation, promoting nerve repair. Thus, skin neuroregeneration may be almost completely inhibited by the lack of angiogenesis at the grafting site [138]. Based on these observations, it was suggested that optimal artificial skin graft for effective sensory nerve regeneration should inhibit the proliferation and accumulation of fibroblasts and myofibroblasts around nerve endings as well as should have optimal porosity and biodegradability supporting new blood vessel formation [150,151].

Only very few attempts have been undertaken to reconstruct skin nerves using artificial skin grafts pre-seeded with various adult stem cells or Schwann cells. Wang et al. [152] developed tissue-engineered nerve conduits by seeding iPSC-derived neural crest stem cells (NCSCs) into nanofibrous tubular scaffolds. The implantation of tissue-engineered nerve conduits resulted in an accelerated regeneration of sciatic nerves in a rat model. Moreover, the transplanted NCSCs had the ability to differentiate toward Schwann cells and promote axonal myelination. Thus, this technique could be potentially translated to the reconstruction of skin nerves using artificial skin substitutes. Blais et al. [153] demonstrated nerve function recovery in vivo in a mouse model after transplantation of collagen/chitosan sponge pre-seeded with human skin fibroblasts, human skin keratinocytes, and murine Schwann cells. Importantly, they observed that artificial skin graft containing Schwann cells showed a current perception threshold comparable to normal skin.

#### 3.2.5. Commercially Available Skin Grafts

There are many commercially available acellular and cellular artificial skin substitutes, which were designed for specific clinical use, including the treatment of chronic and burn wounds. Biobrane^®^ is an acellular dermo-epidermal skin substitute dedicated for the treatment of partial- and full-thickness burns in children, made of porcine type I collagen, packing an inner dermal layer of a 3D nylon filament that is also partially imbedded in an outer epidermal layer made of an ultrathin silicone film. The procedure of Biobrane^®^ grafting is relatively simple; however, this skin graft is often intolerant to contaminated wound beds [2,47]. TransCyte^®^ is a cellular dermo-epidermal graft for partial- and full-thickness burns treatment, composed of a synthetic polymeric membrane as epidermal layer and human neonatal fibroblasts cultured on a scaffold made of porcine type I collagen, which is coated with bio-absorbable polyglactin. Although TransCyte^®^ is characterized by good availability and easy storage, it may be used only as temporary solution [54]. Hyalomatrix^®^ is a bilayered acellular dermo-epidermal substitute for burns and chronic wounds treatment, composed of the HA-based biomaterial with a temporary external epidermal layer made of silicone [55]. Matriderm^®^ is an acellular dermal graft for full-thickness burns and chronic wounds treatment, made of bovine type I collagen and elastin [156,157,158,159,160]. Dermagraft^®^ is a cellular dermal graft used for the treatment of burns and chronic wounds (including chronic diabetic foot ulcers), composed of human neonatal fibroblasts plated onto a bioresorbable polyglactin mesh scaffold. The use of Dermagraft^®^ carries low risk of rejection, but due to its weak ECM structure, it may cause some complications like infections [161]. BioSeed^®^ is a cellular epidermal skin substitute designed for the treatment of chronic leg ulcers, prepared by culturing autologous keratinocytes in a fibrin sealant [162]. Laserskin^®^ is a thin and transparent cellular epidermal graft dedicated for the treatment of burn wounds or chronic full-thickness ulcers, made of benzyl esterified hyaluronan derivative with autologous keratinocytes cultured on its surface [163]. Other examples of commercially available skin grafts are presented in Table 4.

## 4. Concluding Remarks

The ideal artificial skin graft for chronic wound treatment should provide a barrier against pathogens, maintain optimal moisture at the wound-biomaterial interface, supply oxygen (by being oxygen permeable), and primarily promote the skin healing process. Skin substitutes should also be flexible and easily adjustable to the wound bed in order to provide “intimate contact” (without “dead spaces”), facilitating skin regeneration and reducing risk of infections. Treatment of chronic wounds is a big challenge since skin regeneration is hindered by alkaline pH (≥ 7.15) occurring at the wound site, which is optimal for MMP activity and bacteria growth. Therefore, skin graft for chronic wound therapy should also have the ability to lower the pH value (below 7.0), which is known to significantly inhibit MMP activity, prevent infections, and stimulate fibroblast proliferation [53,179]. Ideal artificial skin graft would also reconstruct skin appendages (hairs, sweat glands), pigmentation, and nerves to provide aesthetic appearance of the healed wound and to recover skin sensory and thermoregulatory functions. Taking into account the above-mentioned features of skin substitutes, the production of an ideal artificial graft seems to be a very complex, time-consuming, and costly process. It is therefore not surprising that there are no reports in the available literature on a universal tissue engineered product that would fulfill all requirements of the ideal skin graft. Thus, a rational approach is to produce an artificial skin substitute that is patient-specific and primarily tailored to actual needs.

**Figure 7:**
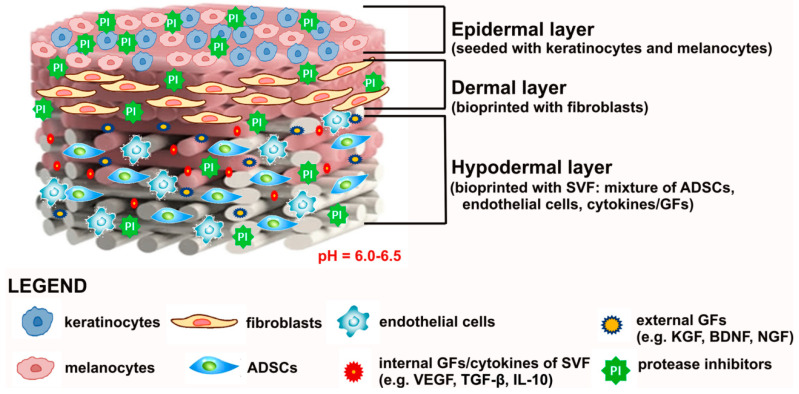
Graphical representation of the potentially ideal artificial skin graft.

Nevertheless, due to recent progress in the field of materials science, development of artificial skin grafts that would address all features of the ideal transplant is hypothetically possible. Ideal skin grafts may be produced as multimaterial scaffolds by a combination of additive manufacturing (e.g., 3D bioprinting) with electrospinning. By combining these two techniques, it is possible to generate biomaterial with gradually changing porosity, mimicking nanostructures of the skin ECM [180,181]. The ideal skin graft would be produced as a trilayered hydrogel biomaterial. Hydrogels have a form of a highly hydrated interconnected matrix, which is highly absorbent and may contain 99% of biological fluids [134,182]. Thus, hydrogel biomaterials made of natural polymers (collagen, fibrin, chitosan, or HA) or their composites with synthetic materials would not only provide appropriate moisture and oxygen supply at the wound site, but would also be perfect for encapsulation of the cells and GFs. The porous hypodermal layer of the ideal skin graft could be bioprinted with adipose tissue-derived SVF, which is a mixture of anti-inflammatory cytokines, GFs as well as various mature and stem cells (fibroblasts, muscle cells, endothelial cells, ADSCs, pericytes, hematopoietic stem cells) [41,42,43]. Therefore, the SVF-enriched hypodermal layer would have immunomodulatory properties and the ability to promote skin regeneration and vascularization. In turn, good skin vascularization would promote neuroregeneration [138]. Incorporation of additional GFs (e.g., KGF, brain-derived neurotrophic factor–BDNF, nerve growth factor–NGF) within the hypodermal layer of the graft could also induce transdifferentiation of ADSCs toward sweat glands and nerves. Importantly, ADSCs and SVF have also been reported to be effective in hair follicle regeneration, inducing hair growth [183]. The dermal layer of the skin graft would be bioprinted with fibroblasts as a highly porous structure to ensure new blood vessel formation and nutrient supply to the wound bed. Whereas, the epidermal layer would have a form of thin, elastic, and oxygen permeable hydrogel membrane acting as a barrier against pathogens. The membrane would be pre-seeded with keratinocytes and melanocytes to ensure accelerated re-epithelialization of the wound and normal pigmentation, respectively. To provide efficient healing of chronic wounds, artificial skin graft may have also protease inhibitors incorporated and pH in the range of 6.0–6.5, which would inhibit MMP activity at the wound site without exerting a negative effect on viability of cells incorporated within the artificial graft (pH below 6.0 may reduce cell viability) [53]. Figure 7 presents a graphical model of potentially ideal artificial skin graft for chronic wound treatment.

However, as was mentioned previously, the production of such ideal skin graft is time-consuming and costly. Therefore, although there are many newly developed and commercially available artificial skin grafts, it is hard to find bioengineered skin substitutes that would have the ability to completely replicate skin and to significantly reduce inflammation in order to reverse the chronicity of the wounds [46]. Moreover, application of currently available artificial skin grafts has some limitations, like low porosity resulting in reduced vascularization, poor mechanical integrity, skin integration failure, and unaesthetic scarring [2,46]. Furthermore, bioengineered cellular skin substitutes are commonly produced using fibroblasts and keratinocytes, making it impossible to reconstruct appropriate skin vascularization, pigmentation, hair, and sweat glands. This problem may be overcome by generation of bioengineered skin graft with the use of additional cell types like endothelial cells, melanocytes, hair follicle stem cells, and MSCs [49,107,141,147,184,185]. Vascularization of the grafts can be improved by loading of the artificial skin constructs with either MSCs or SVF [7,44,45], endothelial cells [184,186] or angiogenic factors (e.g., VEGF, PDGF) [187]. The incorporation of undifferentiated MSCs into the matrix of the skin grafts has also been proven to significantly reduce scarring during the wound healing process [7,188]. Whereas skin appendages may be reconstructed by biomaterial seeding with various types of stem cells (including BMDSCs, ADSCs, epidermal stem cells, dermal stem cells, hair follicle stem cells), sweat gland cells, or dermal papilla cells [138].

Application of bioengineered artificial skin grafts in clinical practice is primarily limited by the expensive and time-consuming production process of cellular substitutes, and their short shelf life. Some of the mentioned limitations may be overcome by the use of advanced production techniques like 3D bioprinting, which allows for patient-customized and on-demand generation of living skin substitutes with either autologous SVF or allogeneic cells (fibroblasts, keratinocytes, MSCs) incorporated [147,189]. Another possibility includes replacement of cellular grafts with acellular skin substitutes, having GFs or other bioactive agents incorporated to promote migration, proliferation, and differentiation of host cells at the wound site. Nevertheless, recent rapid progress in the field of engineering of biomaterials and tissue engineering offers hope for the development of new technology, allowing for fast, personalized, and cost-effective production of functional cell-based artificial skin substitutes capable of restoration of skin appendages, normal pigmentation, and nerves.

## Figures and Tables

**Table 1:** Bioengineered artificial skin grafts made of various natural and synthetic polymers.

Biomaterial Composition	Structural Type of Graft	Cellular Content	Demonstrated Effect In Vitro or In Vivo	Ref.
Collagen, PLGA, glucophage	Dermal	Acellular	Increased collagen content and accelerated healing of diabetic wounds in rats	[82]
Bovine type I collagen, HA	Dermal	Acellular	Improved granulation tissue formation in full-thickness skin defect rat model	[99]
Collagen, alginate, curcumin-loaded chitosan nanoparticles	Dermal	Acellular	Enhanced healing with complete re-epithelialization of diabetic wounds in a rat model	[100]
Gelatin, carboxymethyl chitosan	Dermal	Acellular	Expression of type I collagen and VEGF by mouse embryonic fibroblasts in vitro	[101]
Montmorillonite, bacterial cellulose	Dermal	Acellular	Enhanced wound healing, granulation tissue formation, and re-epithelialization of the burn wounds in murine model	[102]
Carboxymethyl cellulose, PEG	Dermal	Acellular	Accelerated healing of full-thickness wounds in diabetic rats	[103]
Silk fibroin, PLACL, vitamin E, curcumin	Dermal	Acellular	Enhanced fibroblast proliferation and collagen synthesis in vitro	[104]
Fibrin	Dermal	Cellular (fibroblasts)	Promotion of wound bed maturation in diabetic rats	[69]
Collagen, HA, EGF	Dermal	Cellular (fibroblasts)	Increased VEGF and HGF release by fibroblasts in vitro	[105]
PCL, gum tragacanth, curcumin	Dermal	Cellular (MSCs)	Accelerated healing, increased granulation tissue formation, and collagen deposition in diabetic rat model	[106]
Chitosan	Dermal	Cellular (fibroblasts, hair follicle stem cells)	Accelerated full-thickness wound healing in irradiated rats and reduced scarring	[107]
Bovine type I collagen	Dermo-epidermal	Cellular (fibroblasts, keratinocytes)	Formation of epidermis and dermis comparable to native skin in patients with acute partial- or full-thickness skin defects *	[67]
Fibrin-coated poly-l-lactide (PLLA), collagen	Dermo-epidermal	Cellular (fibroblasts, keratinocytes)	Promotion of fibroblast proliferation/migration as well as keratinocyte growth in vitro	[71]
Fibrin	Dermo-epidermal	Cellular (fibroblasts, keratinocytes, ADSCs)	In vitro development of trilayered skin substitute with dermal, epidermal, and hypodermal layer	[75]

* results obtained with clinical trials.

**Table 2:** Nanocomposite biomaterials with antibacterial properties for potential use as artificial skin grafts and wound dressings.

Biomaterial Composition	Type of Nanoparticles	Tested Bacterial Strain	Demonstrated Effect In Vitro or In Vivo	Ref.
Chitosan, HA	nAg	*E. coli*, *S. aureus*, MRSA, *P. aeruginosa*, *K. pneumonia*	Antibacterial activity with low cytotoxicity against human dermal fibroblasts in vitro (at low concentrations of nAg)	[111]
Poly (vinyl alcohol) (PVA) carboxymethyl-chitosan	nAg	*E. coli*	Antibacterial activity in vitro; effect on eukaryotic cells was not tested	[121]
Chitosan, 4-(ethoxycarbonyl) phenyl-1-amino-oxobutanoic acid	nAg	*E. coli*, *S. aureus*, *P. aeruginosa*	Antibacterial activity in vitro; effect on eukaryotic cells was not tested	[122]
Chitosan, PVA	nAg	*E. coli*	Antibacterial activity in vitro; effect on eukaryotic cells was not tested	[123]
Chitosan, gelatin	nAg	*E. coli*, *S. aureus*	Antibacterial activity with no cytotoxic effect against mouse fibroblasts in vitro; accelerated wound healing in a rabbit model	[124]
Biosynthesized bacterial nanocellulose	nAg	*S. aureus*, *P. aeruginosa*, *S. epidermidis*	Antibacterial activity with no cytotoxic effect against human dermal fibroblasts in vitro (at low concentrations of nAg)	[125]
2-acrylamido- 2-methylpropane sulfonic acid sodium salt	nAg	MRSA, *P. aeruginosa*	Antibacterial activity with no cytotoxic effect against human dermal fibroblasts in vitro; prevention of bacterial colonization in a porcine burn model	[126]
Sulfonated polystyrene	nAg	*E. coli*, *S. aureus*, *P. aeruginosa*, *S. epidermidis*	Antibacterial activity in vitro; effect on eukaryotic cells was not tested	[127]
Chitosan	nAu	*S. aureus*, *P. aeruginosa*	Bactericidal effect against biofilm forming antibiotic resistant strains with no cytotoxic effect against human dermal keratinocytes in vitro	[128]
Alginate, polyethylene glycol diacrylate	Cu-doped bioactive glass nanoparticles	*E. coli*, *S. aureus*	Antibacterial activity in vitro; accelerated collagen deposition and promoted early angiogenesis of diabetic full-thickness wounds in a mouse model	[129]
Chitosan-g-pluronic copolymer	nanocurcumin	*E. coli*, *S. aureus*, *P. aeruginosa*, *S. typhimurium*	Antibacterial activity with no cytotoxic effect against human dermal fibroblasts in vitro; enhanced collagen deposition, granulation, and wound maturity of burn wounds in a mouse model	[130]
Chitosan, gelatin	nFe_3_O_4_	*E. coli*, *S. aureus*	Antibacterial activity in vitro; effect on eukaryotic cells was not tested	[131]
2-acrylamido- 2-methylpropane sulfonic acid, acrylamide, acrylonitrile, acrylic acid	nFe_3_O_4_	*E. coli*, *B. subtilis*	Antibacterial activity in vitro; effect on eukaryotic cells was not tested	[132]
Chitosan, pectin	nTiO_2_	*E. coli*, *S. aureus*, *P. aeruginosa*, *B. subtilis*	Antibacterial activity with no cytotoxic effect against mouse fibroblasts in vitro; accelerated wound healing in a rat model	[118]
Chitosan	nTiO_2_	*S. aureus*	Bactericidal effect in vitro; accelerated wound healing in a rat model	[119]
Chitosan, ECM sheet from human adipose tissue	nTiO_2_	*E. coli*, *S. aureus*	Antibacterial activity in vitro; promotion of granulation tissue formation, re-epithelialization, and angiogenesis in a rat model	[120]
Chitosan, poly(*N*-vinylpyrrolidone)	nTiO_2_	*E. coli*, *S. aureus*, *P. aeruginosa*, *B. subtilis*	Antibacterial activity with no cytotoxic effect against mouse fibroblasts in vitro; accelerated healing of open excision type wounds in a rat model	[133]
Poly(*N*-vinylpyrrolidone)	nTiO_2_	*S. aureus*	Antibacterial activity in vitro; effect on eukaryotic cells was not tested	[134]
Chiotsan	nZnO	*P. aeruginosa*, *S. intermedius*, *S. hyicus*	Antibacterial activity with no cytotoxic effect against human dermal fibroblasts in vitro; accelerated wound healing and faster re-epithelialization in a rat model	[116]
Bacterial cellulose	nZnO	*E. coli*, *S. aureus*, *P. aeruginosa*, *C. freundii*	Antibacterial activity in vitro; accelerated wound healing in a burn mouse model	[135]
Alginate	nZnO	*E. coli*, *S. aureus*, MRSA	Antibacterial activity with low cytotoxicity against human dermal fibroblasts in vitro (at low concentrations of nZnO); re-epithelialization in ex-vivo porcine skin model	[136]
Chitosan, alginate	nZnO	*S. aureus*	Antibacterial activity with low cytotoxicity against human cervical cancer cells (HeLa cell line) in vitro; improved tissue generation and accelerated wound healing in a mouse model	[137]

*Bacillus subtilis*: *B. subtilis*; *Citrobacter freundii*: *C. freundii*; *Escherichia coli*: *E. coli*; *Klebsiella pneumonia*: *K. pneumonia*; *Pseudomonas aeruginosa*: *P. aeruginosa*; *Staphylococcus aureus*: *S. aureus*; methicillin-resistant *Staphylococcus aureus*: MRSA; *Staphylococcus epidermidis*: *S. epidermidis*; *Staphylococcus hyicus*: *S. hyicus*; *Staphylococcus intermedius*: *S. intermedius*; *Salmonella typhimurium*: *S. typhimurium*.

**Table 3:** Bioengineered artificial skin grafts designed for the reconstruction of skin appendages, pigmentation, and nerves.

Biomaterial Composition	Skin Appendage or Pigmentation or Nerves	Cellular Content	Demonstrated Effect In Vitro or In Vivo	Ref.
Chitosan	Hair follicles	Human fibroblasts and hair follicle stem cells	Accelerated healing and the presence of viable follicle stem cells in the irradiated wound of rats	[107]
Collagen, glycosaminoglycan	Hair follicles	Murine dermal papilla cells, human fibroblasts and keratinocytes	Successful generation of chimeric hair follicles in an athymic nude mouse model	[142]
Type I collagen	Hair follicles	Human neonatal dermal keratinocytes and fibroblasts, human dermal papilla cells	Differentiation of human keratinocytes into hair follicle lineage in vitro; hair growth after 4-6 weeks in an athymic nude mouse model	[143]
Bovine type I collagen, chondroitin-6-sulfate (Integra^®^)	Hair follicles	Murine newborn epidermal and dermal stem cells	Reconstruction of skin with proper proportions and topological organization, showing large amount of hair follicles (in vivo mouse model)	[154]
Matrigel™ matrix	Sweat glands	Human eccrine sweat gland cells	Formation of the 3D structures in vitro, resembling the morphology of eccrine sweat glands	[144]
Matrigel™ matrix	Sweat glands	Human eccrine sweat gland cells	Reconstruction of tubular-like structures in athymic nude mice (20% of the de novo formed tubular-like structures were coils and 80% were ducts)	[145]
Matrigel™ matrix mixed (1:2) with type I collagen, EGF-loaded gelatin microspheres	Sweat glands	Human skin fibroblasts and keratinocytes, human sweat gland cells	Formation of sweat gland-like structure in vitro; accelerated regeneration of full-thickness cutaneous wounds in an athymic mouse model	[146]
Matrigel™ matrix	Sweat glands	Human eccrine sweat gland cells	Formation of the 3D sweat gland-like structures under in vitro conditions	[155]
Collagen	Pigmentation	Human skin fibroblasts and keratinocytes, human melanocytes	Uniform skin pigmentation; tissue organization resembling native skin	[147]
PLACL, poly(propylene glycol)	Nerves	iPSC-derived neural crest stem cells	Regeneration of sciatic nerves and promoted axonal myelination in a rat model	[152]
Collagen, chitosan	Nerves	Human skin fibroblasts and keratinocytes, murine Schwann cells	Enhanced nerve migration and promoted myelin sheath formation in vitro; nerve function recovery in an athymic nude mouse model	[153]

**Table 4:** Commercially available skin grafts used in the treatment of chronic wounds.

Commercial Product	Biomaterial Composition	Structural Type of Graft	Cellular Content	Indications for Use	Ref.
Epigard^®^	Polytetrafluorethylen, polyurethane	Epidermal	Acellular	Preparation of wound bed before skin transplantation	[160,164]
JACE^®^	No biomaterial used (autologous keratinocyte sheet prepared based on Green method)	Epidermal	Cellular (keratinocytes)	Full-thickness skin defects, extensive burn wounds	[165,166,167]
MySkin™	Silicone	Epidermal	Cellular (keratinocytes)	Diabetic foot ulcers	[168]
Insuregraf^®^	Porcine type I collagen	Dermal	Acellular	Burn wounds	[169]
Integra^®^	Bovine type I collagen, chondroitin-6-sulfate	Dermal	Acellular	Partial- and full-thickness burns, chronic ulcers	[157,170]
Nevelia^®^	Calf type I collagen	Dermal	Acellular	Burn wounds	[171]
Hyalograft 3D^®^	HA	Dermal	Cellular (fibroblasts)	Deep burns, foot ulcers	[172]
PELNAC™	Porcine atelocollagen, silicone	Dermo-epidermal	Acellular	Partial- and full-thickness wounds, large acute burns	[173,174]
Apligraf^®^	Bovine type I collagen	Dermo-epidermal	Cellular (fibroblasts, keratinocytes)	Partial- and full-thickness burns, chronic ulcers	[175,176]
MyDerm™	Fibrin	Dermo-epidermal	Cellular (fibroblasts, keratinocyte)	Full-thickness wounds	[177,178]
